# Electroencephalography Signal Analysis for Human Activities Classification: A Solution Based on Machine Learning and Motor Imagery

**DOI:** 10.3390/s23094277

**Published:** 2023-04-26

**Authors:** Tarciana C. de Brito Guerra, Taline Nóbrega, Edgard Morya, Allan de M. Martins, Vicente A. de Sousa

**Affiliations:** 1 Graduate Program in Electrical and Computer Engineering (PPgEEC), Federal University of Rio Grande do Norte, Natal 59078-970, Brazil; 2 Graduate Program in Neuroengineering, Edmond and Lily Safra International Institute of Neuroscience, Santos Dumont Institute, Macaíba 59280-000, Brazil

**Keywords:** EEG, machine learning, random forest, motor imagery, mindwave, V-AMP

## Abstract

Electroencephalography (EEG) is a fundamental tool for understanding the brain’s electrical activity related to human motor activities. Brain-Computer Interface (BCI) uses such electrical activity to develop assistive technologies, especially those directed at people with physical disabilities. However, extracting signal features and patterns is still complex, sometimes delegated to machine learning (ML) algorithms. Therefore, this work aims to develop a ML based on the Random Forest algorithm to classify EEG signals from subjects performing real and imagery motor activities. The interpretation and correct classification of EEG signals allow the development of tools controlled by cognitive processes. We evaluated our ML Random Forest algorithm using a consumer and a research-grade EEG system. Random Forest efficiently distinguishes imagery and real activities and defines the related body part, even with consumer-grade EEG. However, interpersonal variability of the EEG signals negatively affects the classification process.

## 1. Introduction

The World Health Organization (WHO) published the Global Disability Action Plan in 2015 [1]. The document presents statistical data and establishes objectives and action plans to promote quality of life for people with some disabilities. The main goals involve removing barriers and improving access to health services and programs. People with disabilities represent about 15% of the world population, which will increase due to longer life expectancy and continuous technological advances [1].

The development of assistive technologies promotes accessibility and rehabilitation [2]. The WHO on Disability [2] suggests research actions for developing technological devices to provide accessibility and rehabilitation. These devices could be a simple crutch, cane, or even a more complex solution, such as Brain-Computer Interface (BCI).

The advances in neuroscience associated with digital signal processing and biomedical instrumentation allow a better understanding of the brain’s functions [3]. These studies enable the development of systems capable of translating biological signals into digital responses [4]. Hans Berger recorded the first brain electrical activity from a human scalp [5]. Years later, Grey Walter described the first BCI. He implanted electrodes directly into the motor cortex and recorded brain activity. The results showed the possibility of predicting body movement by analyzing the brain’s signal [5,6].

The results of these researches stimulated interest in BCI, as they led to the possibility of implementing systems capable of helping impaired people, such as people with quadriplegia and some degenerative disease, for example, Amyotrophic Lateral Sclerosis (ALS) [7]. BCI is also used in other proposals, such as the control of Unmanned Aerial Vehicles (UAV) [8]. Therefore, its relevance is not limited to applications for people with physical and motor limitations [9].

BCI systems use electroencephalogram (EEG) to acquire brain electrical activity through non-invasive electrodes and safe and low-cost procedures [3,7]. This method has an excellent temporal resolution, allowing applications in many fields [10].

Extracting information robustly and reliably from EEG signals can be challenging [11]. Most projects generally consider the properties of EEG signals in the time, frequency, and space domains [12]. The objective is to identify the features of the signals associated with the brain activity of interest so that it is possible to control a BCI based on the user’s intentions. Thus, processing these signals considers two fundamental steps: feature extraction and classification [3].

Another problem involving BCI is rapid data manipulation; the user must experience real-time interactions that mimic the body’s natural actions [13]. The natural interaction with the external environment occurs from communicating the Central Nervous System (CNS) with the muscles through the Peripheral Nervous System (PNS). This communication is efferent—from CNS to PNS (control of voluntary movement); and afferent—from the sensory system to the CNS (sensation and perception). Any damage to these pathways can result in sensorimotor problems and permanent damage to motor control [9].

EEG is widely used to control BCI. However, these signals are very susceptible to noise, and pattern identification is complex [13]. In this context, it is possible to use data analysis to interpret these signals adequately. Studies show that machine learning algorithms efficiently explore and classify neural activities [14]. These algorithms automatically classify the data, allowing more practical applications, and it is less dependent on trained professionals [14].

The acquisition protocols used to collect EEG signals usually involve motor tasks, sensory stimuli, such as image and sound presentation, and activities that comprise mental simulation of a motor act, also known as motor imagery [9]. The protocol depends on the application of interest. BCI implemented for people with some motor disability generally aims to implement acquisition protocols independent of motor control. The use of motor imagery makes it possible, for example, for a person with paraplegia to control a neuroprosthesis since the signal to be collected does not depend on the remaining muscle activity [15].

Thus, applications with this type of protocol not only benefit the control of devices through EEG signals but also allow the rehabilitation of people with motor disabilities [15]. This type of rehabilitation is possible due to neuronal plasticity, which is the ability of neurons to establish new connections from new stimuli, which may come from the organism itself or the external environment [7]. The brain can learn new functions and adapt itself, even when some regions have functional impairment [15].

The rest of this paper is structured as follows. Section 2 shows the research background and the related works, while Section 3 focuses on our proposed solution, including the details of signal processing and Machine Learning (ML) structure. Section 4 gives information on the experiment setup and the measurement protocol, and Section 5 shows the results. Finally, Section 6 describes our conclusions and final remarks.

## 2. Related Works and Research Background

The technological evolution of the last decades allowed the development of algorithms capable of solving highly complex tasks, such as those related to EEG signal processing. The information from such signals benefits several applications, not restricted to people with motor limitations. The control of BCI from interpreting and analyzing these signals has aroused increasing interest in the scientific community. Machine learning studies to solve paradigms involving BCI and EEG have increased classification efficiency. The challenge is to develop algorithms capable of providing high reliability and slight response delay concerning pattern recognition of EEG data.

Amin et al. [16] pointed out some challenges inherent to applications involving EEG signal decoding of motor imagery tasks. They proposed the fusion of several models of Convolutional Neural Network (CNN). The method considered multi-layer CNNs with different architectures and characteristics to improve classification accuracy. Also studying EEG signals in applications with motor imagery, Sadiq et al. [17] developed a signal decomposition method based on empirical Wavelet Transform. The study involved the analysis of 18 electrodes to investigate the non-stationary and non-linear behavior of EEG signals, considering the power spectral density and the Hilbert Transform. The researchers considered the power spectral density and the Hilbert Transform. The classification accuracy was 95.2 %, and could be better by incorporating higher-order statistical resources, such as kurtosis.

Craik et al. [14] presented a systematic literature review involving task classification using electroencephalographic signals with machine learning tools. The research revealed that approximately 40% of tasks with motor imagery protocols used CNN as learning architecture. However, the research did not show the use of Random Forest as a classification technique. Liu et al. [18] used Random Forest with motor actions of the lower limbs, aiming to describe the impact of different feedback modalities of the BCI system based on EEG signals that decode extension and flexion of the legs. The results considered real-time simulations and presented average accuracy of 62.33% and 63.89% for two sessions.

Lazurenkoa et al. [19], and Meziani et al. [20] analyzed EEG signals from motor imagery activities without Random Forest, but compared the results with algorithms from Random Forest. In [19], they developed a neural network network strategy to detect EEG signal patterns during motor imagery activities. They compared the results obtained with the Random Forest algorithms, linear discriminant analysis, and linear regression methods considering radial basis functions. The solution presented satisfactory results compared to other algorithms with 82.5% accuracy in the classification. [20] used quantile regression and regularization of the L1 norm to estimate the spectrum of the EEG signal related to imaging tasks. The study also compared the results with other classification algorithms, including Random Forest. None of the works analyzed in this theoretical framework compared motor imagery EEG signals with machine learning. One of the objectives of this paper is to evaluate the performance of a consumer-grade EEG sensor (Mindwave, Neurosky) in an application involving motor imagery.

Consumer-grade and low-cost systems for acquiring EEG signals are in BCI research applications [21]. The authors of [22] used the device Mindwave in two applications: controlling the movements of a robot back and forth and controlling electronic devices. In [23], the target device was Neurosky, with an accuracy of around 95% in controlling a wheelchair using measures of attention, meditation level, and blinking identification. Still, in the context of wheelchair control using Mindwave, [24] researchers used neural networks, especially the Backpropagation algorithm, to predict the direction of an electric wheelchair using the EEG signal of a person with motor limitations. The results presented indicate a correlation coefficient of 0.92804, showing that the accuracy achieved was satisfactory.

Searching for articles that compare consumer-grade EEG sensors with motor imagery-based machine learning algorithms has yet to find published papers. Additionally, Web of Science, Scopus, and PubMed databases did not present research using Random Forest as the primary classification algorithm. Also, most papers used EEG data from the BCI Competition [25]. This is a database by the Berlin Brain-Computer Interface project, a partnership between the USA and Europe to provide systems that allow a direct dialogue between men and machines. Therefore, instead of recording EEG data, most papers use databases. These considerations elucidate the contributions of this paper.

Table 1 summarizes all the research above.

Therefore, to guide the investigation, we propose to discuss the following questions:

- Does ML based on random forest algorithm classify different motor tasks (real or imagery)?
- Does ML based on random forest differentiate real and motor imagery in FP1 electrode?
- What is the classification performance comparing consumer-graded and research EEG devices?
- What is the variability between EEG’s spectro-temporal and spatial distribution characteristics among subjects?

## 3. Proposed Solution

**Figure 1:**
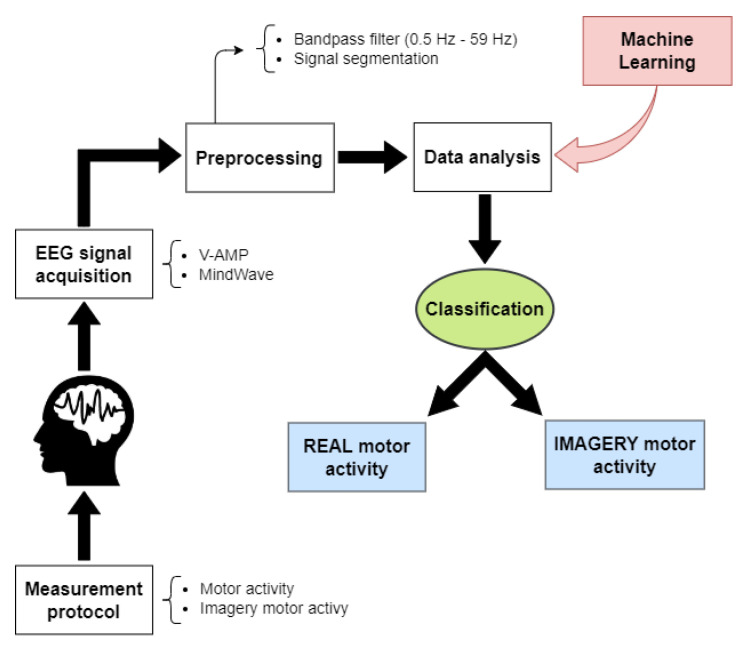
The proposed classification system starts with motor real or imagery tasks. A consumer-graded and a research EEG device simultaneously acquire brain activity. The system preprocesses EEG signals to enter the machine learning algorithms. The expected result is compared to the trial performed by the subject, and the classification identifies the task, motor real or imagery.

In order to be able to distinguish movements based on EEG signs, we propose the classification system presented in Figure 1.

### 3.1. Signal Preprocessing

EEG signals are intrinsically noisy and sensitive to other artifacts, like those originating from blinking and muscular activities [26]. Furthermore, EEG has a high temporal resolution (millisecond scale), but a low spatial resolution (centimeter scale), and their placement also influences the quality of the signal. A diffuse signal’s spatial resolution introduces an additional challenge to identifying and isolating the EEG data task related [14].

Digital filters attenuate noises efficiently [27]. In this proposed classification system we chose a Butterworth band-pass, 3rd order, 0.5 Hz to 59 Hz, filters powerline hum and keeps brain rhythms (delta to low gamma).

**Figure 2:**
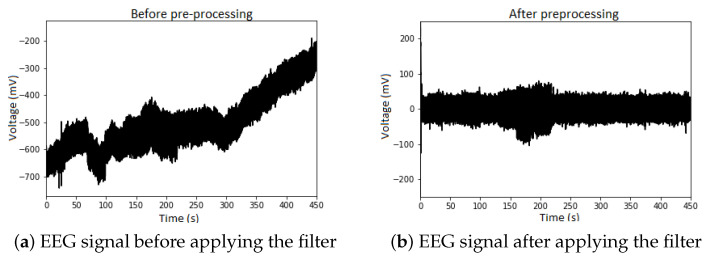
Illustrative EEG signals before and after applying the Butterworth band pass filter. The baseline drift of the signal before preprocessing is evident (**a**) and after removing the drift (**b**).

Another characteristic of the EEG signal is the baseline drift, usually caused by eye movements, deep breaths, and facial muscle movements [28]. Figure 2 shows the signal before the preprocessing with a noticeable drift (Figure 2a), and drift filtered out (Figure 2b).

Participants perform each task for 30 s (motor real or imagery), and the system discards the first 5 s and last 5 s, using 20 s in between, as further explained in Section 4.

### 3.2. Machine Learning

ML is a set of data analysis method that are able to fit very complex models to a dataset using algorithms some time classified as artificial intelligence. This artificial intelligence algorithms acquires knowledge by extracting patterns from raw data, enabling it to execute complex tasks with minimal human intervention [29].

ML algorithms can solve many types of tasks, including classification and regression. In the first one, the algorithm has to specify to which category some input belongs. In the last one, it has to predict a numerical value given some input parameters [29]. Object recognition and securities’ price predictions are examples of, respectively, classification and regression tasks.

Moreover, those algorithms can use three learning processes: supervised, reinforcement, and unsupervised learning. Supervised learning consists of learning from a training set of labeled examples provided by a “supervisor” during a training phase. Each example has a set of inputs and the correct output (or “label”), indicating how the system should behave in that specific case. The goal is for the algorithm to generalize its responses to produce the correct output for sets of inputs not present in the training examples [30].

Reinforcement learning also aims to find the best output to a set of inputs. However, instead of a label, the system provides the algorithm with feedback that shows how good the choice was, but not if it is the best. On the other hand, unsupervised learning comprises finding structures hidden in unlabeled data [30].

This work uses a supervised classification ML algorithm called Random Forest. A Random Forest consists of an ensemble of Decision Trees, a non-parametric ML algorithm, typically grown using the Classification and Regression Tree method [31]. For each set of inputs, each Decision Tree generates an output. Then, in the classification problems, the Random Forest chooses the most popular output as its result [32].

The fundamental concept of Random Forests is simple but efficient. This algorithm is based on group knowledge. Many relatively uncorrelated models (trees) working together will overcome any individual models. While a tree might converge to the wrong result, most others will likely choose the correct answer. So the group will classify correctly [33].

### 3.3. Implementation of the Proposed Solution

This research aims to differentiate a set of motor (real and imagery) activities performed by individuals following an established protocol. This protocol, detailed in Section 4, comprises right and left-hands and right and left ankles movements. The proposed solution must be able to differentiate if the individual imagined or moved a limb and which limb it was.

Before applying the machine learning algorithms, we preprocessed the data, as explained in Section 3.1. After, we calculated the statistical moments from order 1 to 10 of the resulting data; those moments serve as features for our ML algorithms. Then, we split the data between train, validation, and test sets. We use the first two sets to make a feature selection and a grid search process, defining inputs and parameters of the Random Forests. Both sets division, feature selection and grid search used the *Scikit-Learn* library of Python 3.6.3.

**Figure 3:**
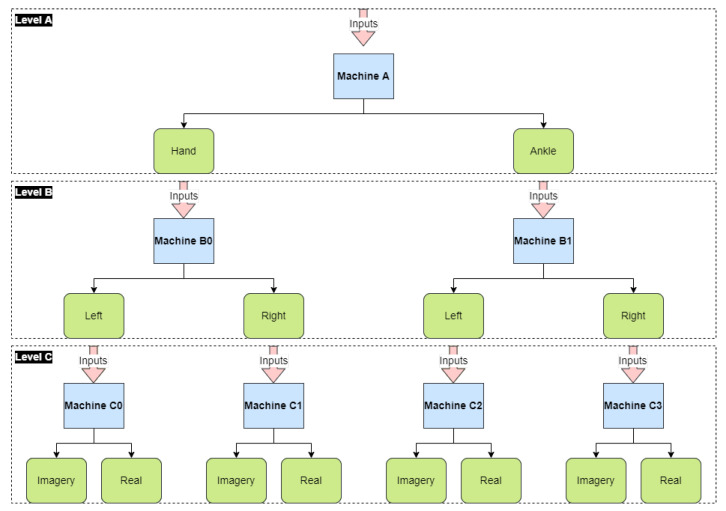
Block diagram of the proposed hierarchical classification architecture.

We divide our classification model into three levels: Level A, Level B, and Level C. Each level is responsible for distinguishing a characteristic of the movement, as the literature suggests that the more specialized the machine, the better its results [33,34]. Each level has the following functionality, as shown in Figure 3:

- **Level A** comprises a single machine responsible for classifying the data according to the region of the movement, i.e., hands or ankles;
- **Level B** contains two machines, one for each region of the movement. The machines at Level B classify the movements between right and left. Considering that, at this point, we have already classified the data by its region, the possible outputs are left-hand, right-hand, left-ankle, and right-ankle movements;
- **Level C** determines whether the subject performed the movement or imagined it. It comprises four machines, each specialized in one possible output of Level B. Therefore, there are eight outputs for Level C: movement of the left-hand, imagery movement of the left-hand, movement of the right-hand, imagery movement of the right-hand, movement of the left ankle, imagery movement of the left ankle, movement of the right ankle, imagery movement of the right ankle.

Hence, after Level C, we have the category split intended.

#### Frameworks’ Definition

**Figure 4:**
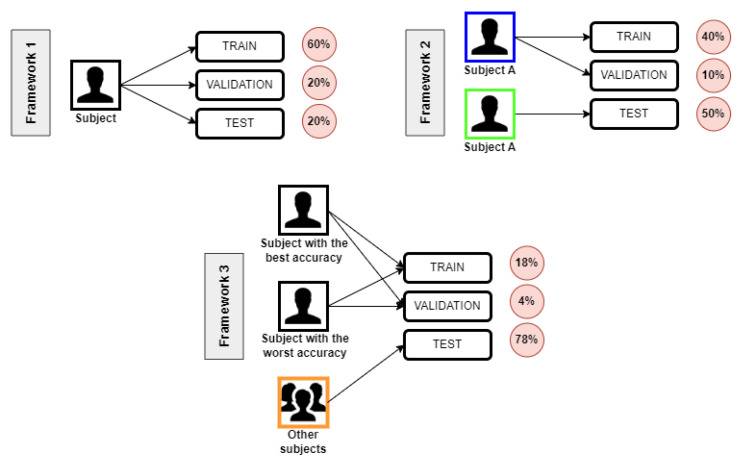
Frameworks used in the research.

Interpersonal variability is a challenge to EEG signals classification, especially when motor imagery is involved, as people tend to imagine or do things in their particular way. Considering that, we defined three frameworks to verify the accuracy of our proposed solution. Each framework splits the data between the train, test, and validation subsets in a particular way, allowing us to evaluate the correlation between EEG signals from different subjects, as shown in Figure 4. Therefore, the proposed solution also aims to identify the difficulty level when we consider different data from different subjects.

The Frameworks differ from one another regarding their way of defining the data from the train, validation, and test subsets, as further explained:

- **Framework 1**: used the same data collection from the same subject to train, validate, and test;
- **Framework 2**: considered two data collections from the same subject and used one of them to train and validate and the other for testing;
- **Framework 3**: to test and validate, we used data collections from the two subjects with the best and the worst accuracy in Framework 1’s classification. Then, we test it with data collections of the remaining seven subjects.

Moreover, concerning the size of the train, test, and validation subsets, the frameworks do as follows:

- **Framework 1**: 60% of data for train, 20% for validation, and 20% for test subsets;
- **Framework 2**: 40% of data for train, 10% for validation, and 50% for test subsets;
- **Framework 3**: 18% of data for train, 4% for validation, and 78% for test subsets.

As the increase in the data variability tends to cause a decrease in accuracy, we expect Framework 1 to have the best results, followed by Framework 2 and then Framework 3. These results would show the uniqueness of each person’s EEG signals, even when performing the same research protocol as others.

Furthermore, it is essential to notice that the choices made for each framework also have implications for their applicability. In Framework 1, the low variability implies the need to train its machines for each use of the subject. Framework 2, however, only needs to train its machines once for each subject. At least, Framework 3, due to its high variability, does not need a training process for each subject or each use. Its machines only need to be trained once to be appropriate for the use of anyone at any time. Thus, there is a clear trade-off between an easier classification and a more straightforward application.

## 4. Experimental Setup and Measurement Protocol

### 4.1. Experimental Setup

**Figure 5:**
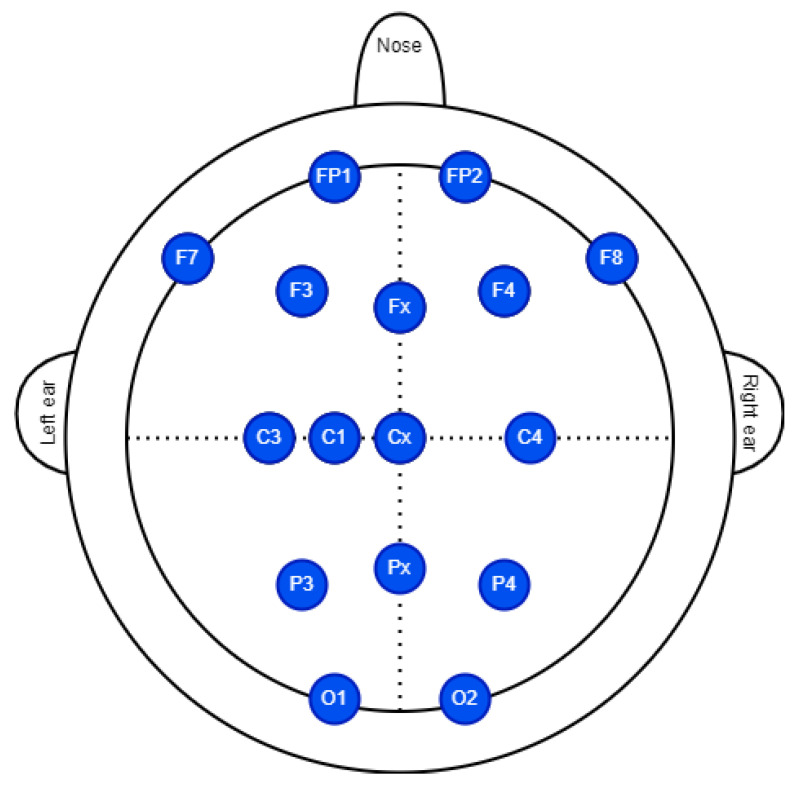
Position of the electrodes we used in the EEG signal aquisition with V-AMP.

The proposal is to analyze EEG signals of individuals submitted to motor imagery tasks. The protocol used two devices: a 16-channel EEG system (V-AMP amplifier) and a single-channel consumer-grade EEG (Mindwave). The V-AMP amplifier (Brain Products GmbH) [35] uses an easycap for positioning 16 active EEG electrodes, using conductive gel to decrease impedance. Figure 5 shows the positioning of the 16 electrodes based on 10–20 electrode system.

**Figure 6:**
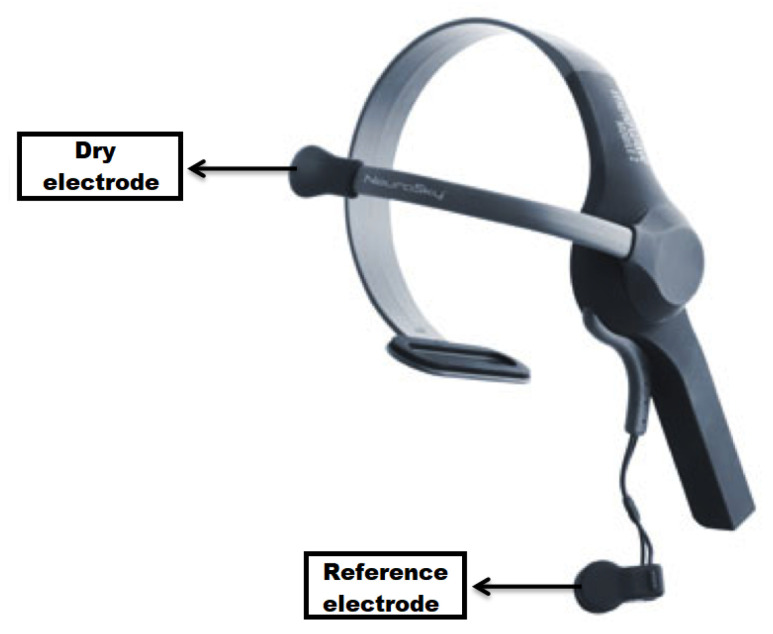
A Mindwave device from the company *Neurosky*. Adapted from [36].

The Mindwave [36], depicted in Figure 6, is a device developed by Neurosky. It consists of a single dry EEG electrode placed in the forehead, above the eyebrow, and it features an ear lobe. It uses Bluetooth to to stream the data.

The software used to acquire the signals from both sensors was OpenVIBE [37]. The data were collected simultaneously. Therefore, it was possible to analyze the same dataset from the two sensors at the same time. The sampling frequency for both is 512 Hz, almost nine times higher than the highest desired signal frequency (59 Hz, see Section 3), respecting Nyquist criterion.

**Figure 7:**
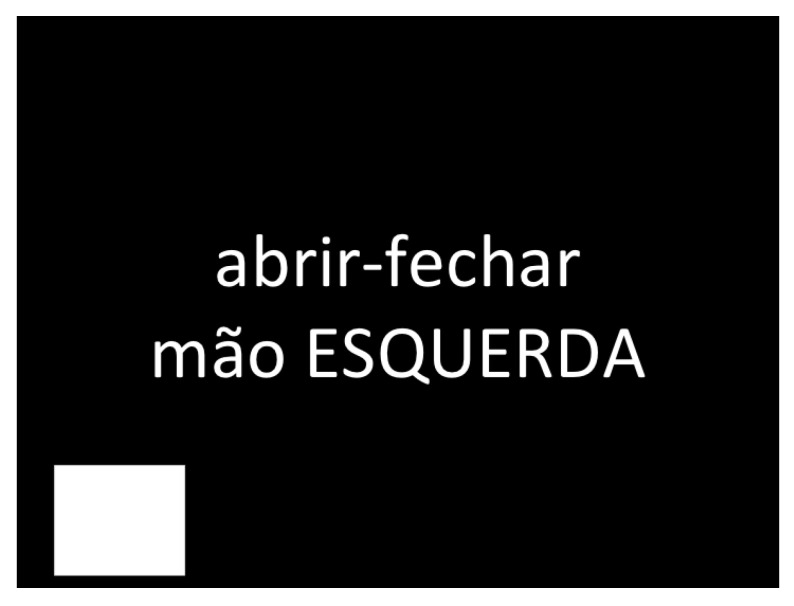
Image presented to the subject with the instruction “open and close the left-hand” in Portuguese. We use the white square in the bottom left to activate the light sensor and indicate the beginning and end of each activity.

**Figure 8:**
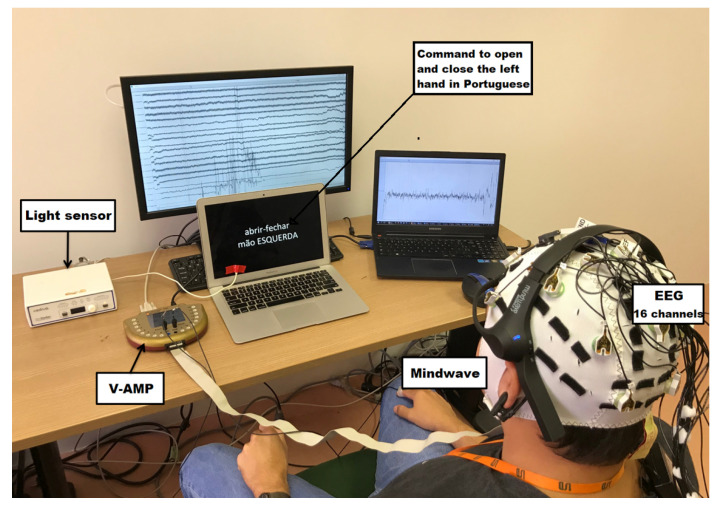
Experimental setup for EEG recording using V-amp and Mindwave, light sensor to synchronize instruction presentation with data recorded.

Another hardware we added to the system was the StimTracker [38] manufactured by Cedrus. This equipment worked as a light sensor. The image presented to the candidate (shown in Figure 7) contains a white square that appears for 0.5 seconds at the beginning and end of each activity. The light sensor is connected to the screen and identifies when the white square appears. The light sensor signal works in parallel with the EEG sensor signals in the OpenVIBE software. Therefore, it is possible to synchronize the EEG signal when the task starts and ends, eliminating the dependence on manual time stamping. Figure 8 shows the equipment layout and collection scenario.

### 4.2. Measurement Protocol

This project was approved by the Local Ethical Committee (CAAE: 79649717.0.0000.5292). The measurement protocol considered 14 healthy participants (10 male and 04 female, aged between 20 and 30 years). The subjects were seated comfortably in a chair and placed their hands on their legs, as illustrated in Figure 8. They executed the sequence of activities presented through a computer screen positioned in front of them, as shown in Figure 7. The set of activities comprised motor and imaginary tasks of the hands and ankles, according to the following task orientation:

- Imagine opening and closing the right-hand;
- Open and close the right-hand;
- Imagine opening and closing the left-hand;
- Open and close left-hand;
- Imagine flexing the right ankle;
- Flex the right ankle;
- Imagine flexing the left ankle;
- Flex the left ankle.

Each task lasted 30 seconds without interval. Each subject performed the protocol twice, resting a few minutes between the recordings. OpenVIBE recorded EEG data from V-amp, Mindwave, and light sensor. EEG signals from V-amp and Mindwave are displayed in real-time on two monitors.

#### Data Selection

Data exclusion attended the following criteria:

- Participants who did not follow the task instruction;
- Data with missing synchronization trigger;
- Low battery of the Mindwave, which impaired data streaming.

Data from 14 participants were recorded, 5 were excluded, and 9 satisfied the system analysis conditions to guarantee reliable results.

## 5. Results and Discussions

### 5.1. Preliminary Analysis

**Figure 9:**
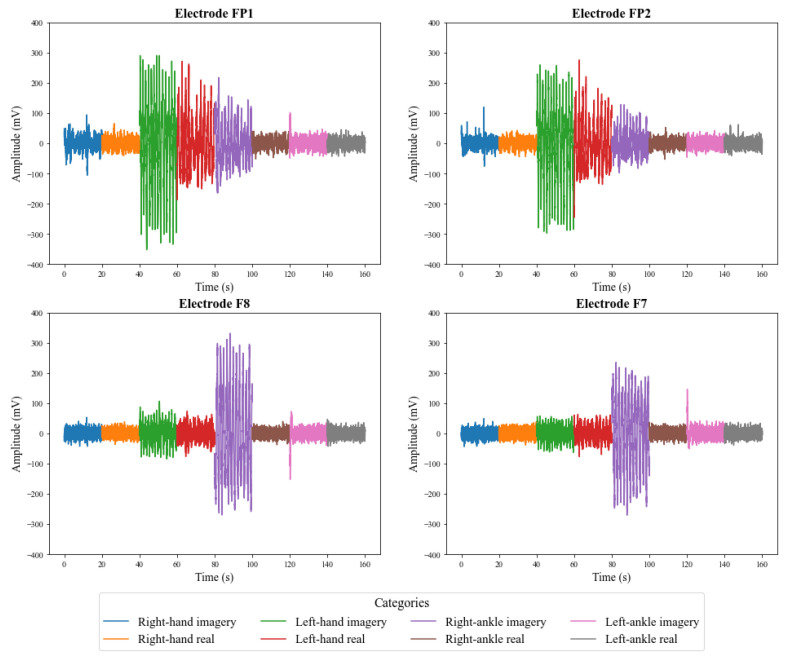
EEG signals captured by the first 4 channels of the sensor V-AMP for **Subject 01**.

**Figure 10:**
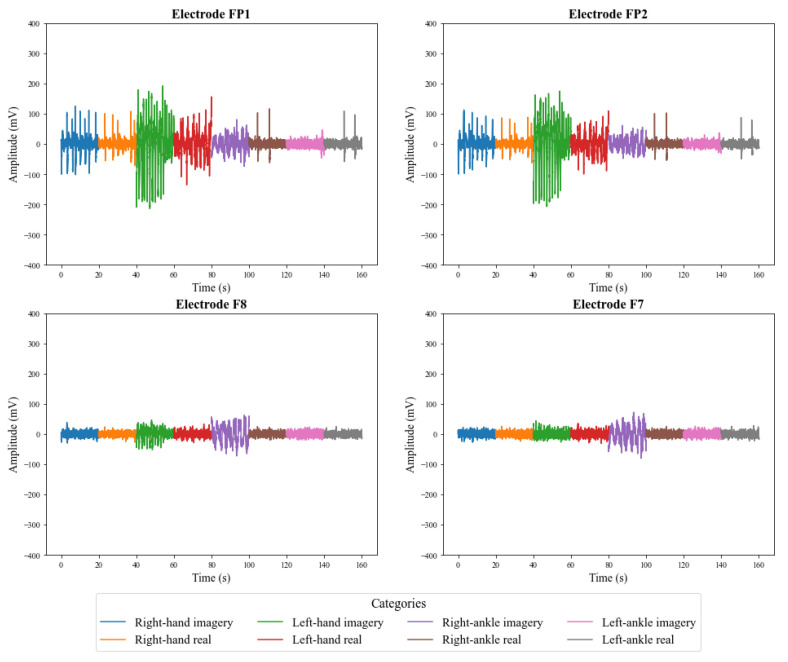
EEG signals captured by the first 4 channels of the sensor V-AMP for **Subject 02**.

**Figure 11:**
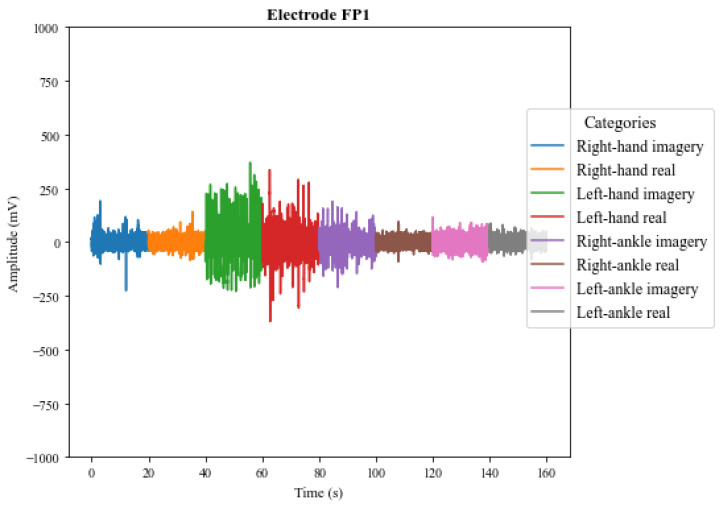
EEG signals captured by the sensor Mindwave for **Subject 01**.

**Figure 12:**
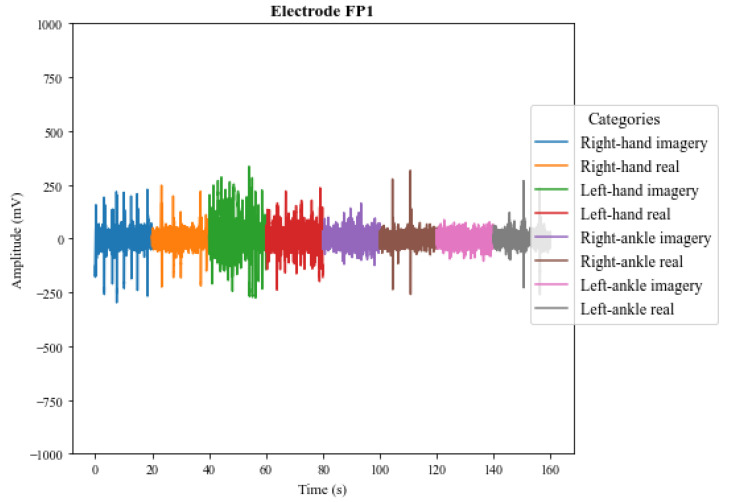
EEG signals captured by the sensor Mindwave for **Subject 02**.

Figure 9 and Figure 10 illustrate the signals captured by the first four channels of the V-AMP sensor for two different subjects. Similarly, Figure 11 and Figure 12 show the signals of the same individuals, in the same collection, by the Mindwave sensor. The graphic presentation of these signals in the referred figures considers the signal strength in time, and a specific color identifies each task. Therefore, it is possible to analyze in time how EEG signal behaves for each person during each activity. The first analysis is that even performing identical tasks, following the same protocol, each person presents unique characteristics in the signal data set.

**Figure 13:**
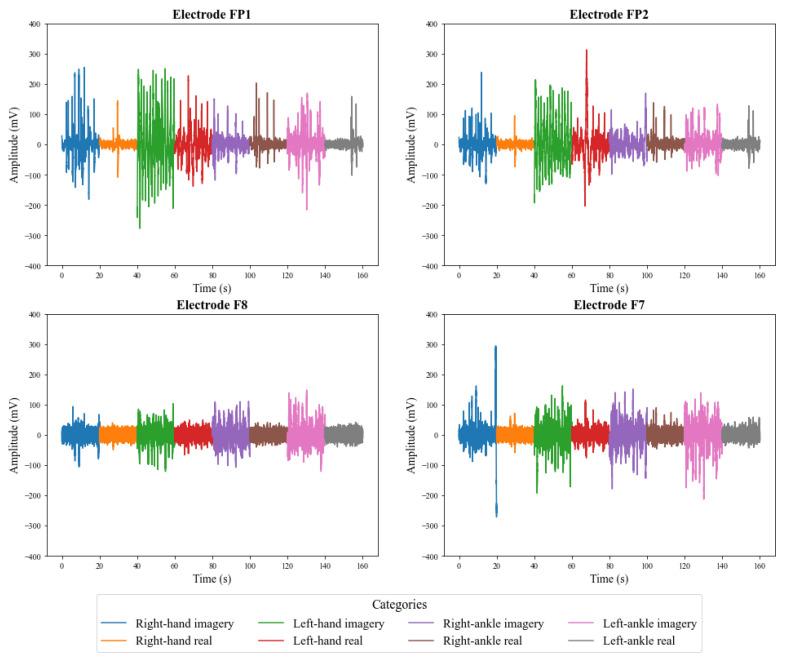
EEG signals captured by the first 4 channels of the sensor V-AMP for the first collect of a subject.

**Figure 14:**
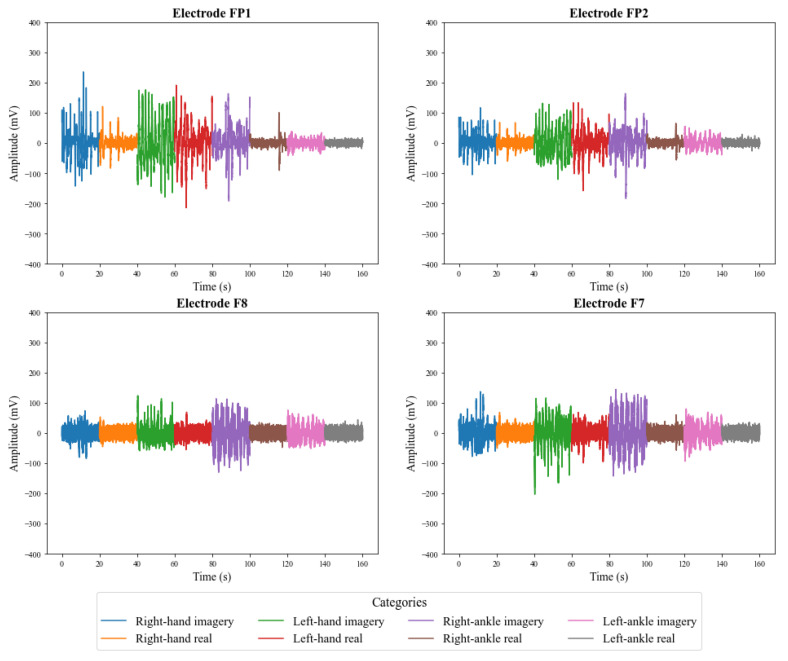
EEG signals captured by the first 4 channels of the sensor V-AMP for the second collect of a subject.

The results also show that in addition to the interpersonal variation, the EEG signals present variations for the same person, even when that person executes the same protocol. The electroencephalographic signal does not have a defined visual pattern for a given action. If the same person performs an activity at a specific time and then repeats the activity at another time, the signal will probably present another visual pattern. Figure 13 and Figure 14 show the signs of the same individual in two different collections with the first four channels of V-AMP. In the images, regardless of which sensor is responsible for the acquisition, the signals are visually different for each recording, and highlight the need of a ML classifier.

### 5.2. Classifier Results Analysis

**Figure 15:**
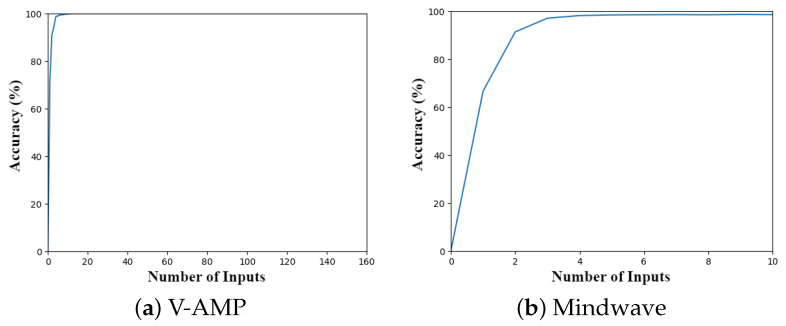
Accuracy of a feature selection for a machine in Level A.

Data processing used machine learning algorithms and Random Forest as a classification tool. One of the steps developed was Feature Selection, which generates better output in the ML algorithm. Figure 15 contemplates the result of the Feature Selection for a given subject, considering the machine learning process at Level A, which classifies into hands or ankles. A controlled amount of inputs in the classification process optimizes the algorithm’s processing, which requires a smaller set of mathematical operations. The results show that the machine needs to use around 5 inputs to reach a satisfactory accuracy, around 100%. Both devices, V-AMP and Mindwave, presented this pattern.

As shown in the diagram in Figure 3, the project comprises the implementation of seven machines learning for the analysis and classification of EEG signals. The machines are divided into levels and specialize in certain classifications. Each machine, at a specific level, implements Feature Selection; this implies that some channels and statistics are more relevant to the classification process for each machine. Table 2 presents the result of the Feature Selection of one of the research participants for V-AMP data.

Mindwave is a single-channel sensor, so the Feature Selection process only considers the moments calculated. Table 3 shows the results of the same individual for the Mindwave data.

We decided to consider the best and worst results to generalize the data exposure of the nine subjects. The metric used was the average of the accuracies of each activity, resulting from the confusion matrix. In other words, the results presented are for the individuals who obtained the highest and lowest average in the activities classification.

#### 5.2.1. Framework 1

**Figure 16:**
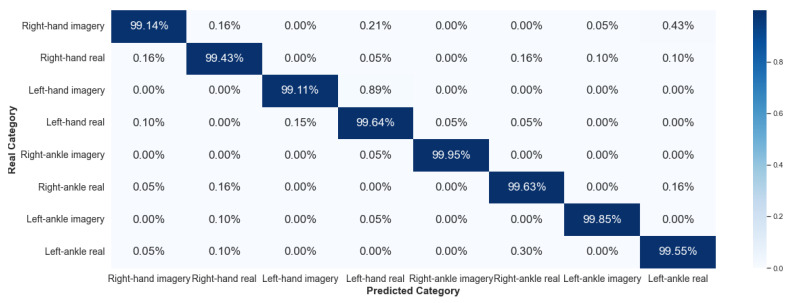
Confusion matrix of Framework 1 using the sensor V-AMP. Subject with the best score.

**Figure 17:**
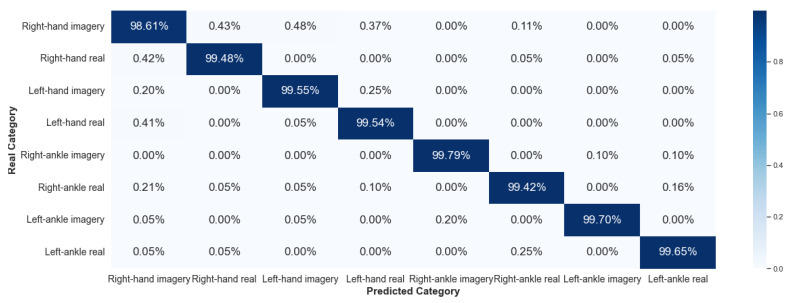
Confusion matrix of Framework 1 using the sensor V-AMP. Subject with the worst score.

**Figure 18:**
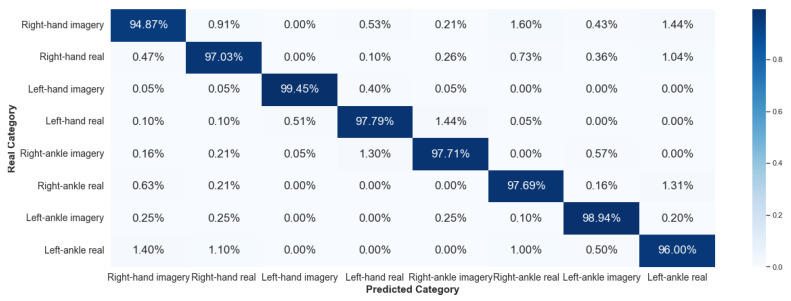
Confusion matrix of Framework 1 using the sensor Mindwave. Subject with the best score.

**Figure 19:**
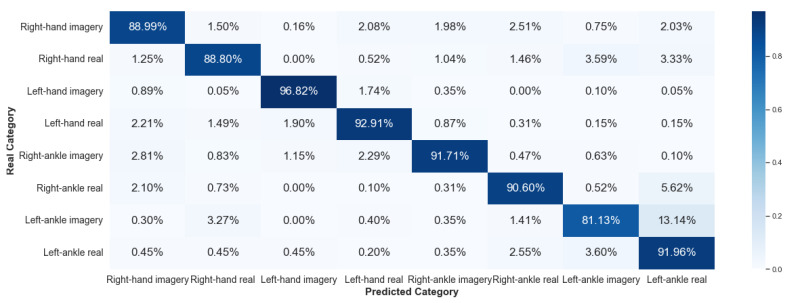
Confusion matrix of Framework 1 using the sensor Mindwave. Subject with the worst score.

Framework 1 considered data from the same individual for all stages of the algorithm: training, validation, and testing. Figure 16 and Figure 17 show the results, via a confusion matrix, for Framework 1 considering V-AMP data for the individual with best and worst performance, respectively. Figure 18 and Figure 19 present the results of the data collected by Mindwave for the same Framework, also considering the best and worst performance.

We expected that this Framework would present a satisfactory result, as this case does not deal with two problems of EEG signal analysis: interpersonal variability and collection of the same individual at different times. Comparing the results of V-AMP with Mindwave indicates that even Mindwave is a simple sensor with a dry electrode and low cost, it can provide a high accuracy rate in the classification process.

Another fact exposed by the results is the stability of the accuracy rate between the worst and the best individual, something expected since the algorithm’s execution happens independently for each person.

#### 5.2.2. Framework 2

**Figure 20:**
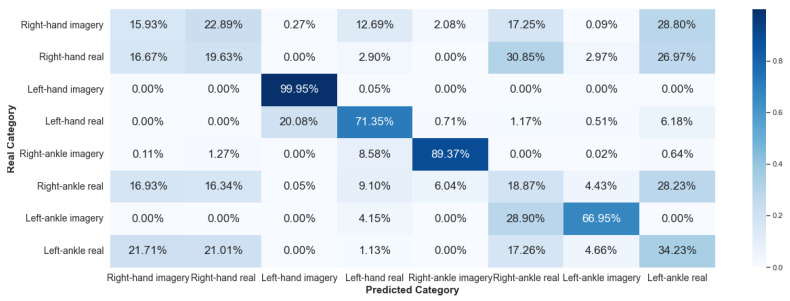
Confusion matrix of Framework 2 using the sensor V-AMP. Subject with the best score.

**Figure 21:**
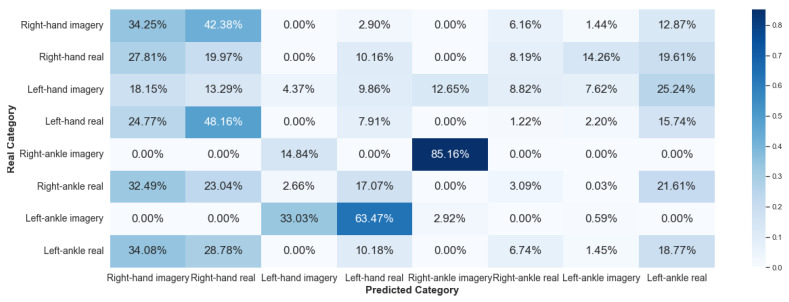
Confusion matrix of Framework 2 using the sensor V-AMP. Subject with the worst score.

**Figure 22:**
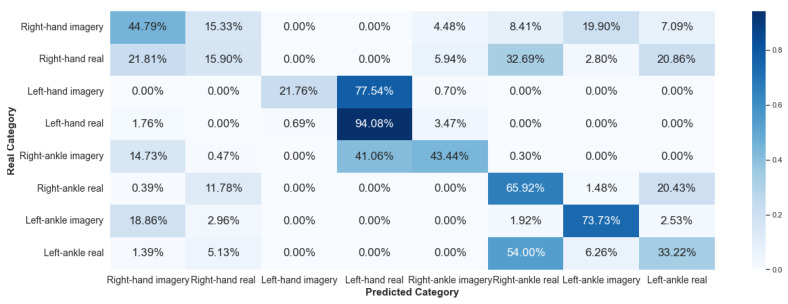
Confusion matrix of Framework 2 using the sensor Mindwave. Subject with the best score.

**Figure 23:**
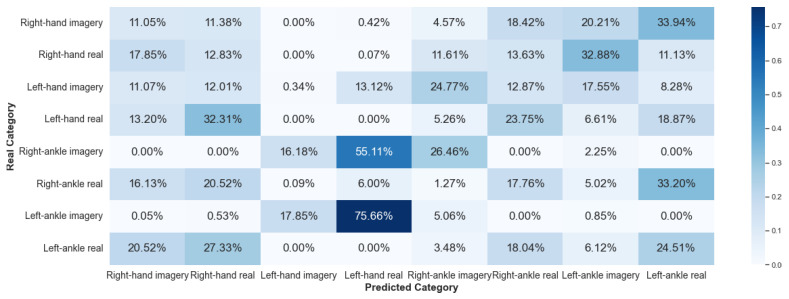
Confusion matrix of Framework 2 using the sensor Mindwave. Subject with the worst score.

Framework 2 uses data from the same subject considering different collections. The results of V-AMP are shown in Figure 20 and Figure 21, and those of Mindwave in Figure 22 and Figure 23.

It is noticeable that compared to Framework 1, the sorting ability of the algorithm for Framework 2 has dropped considerably. This result reaffirms that there is still a significant problem in working with EEG data from different temporal experiences, implying that the classification method developed was inefficient in processing EEG data collected at different times.

#### 5.2.3. Framework 3

**Figure 24:**
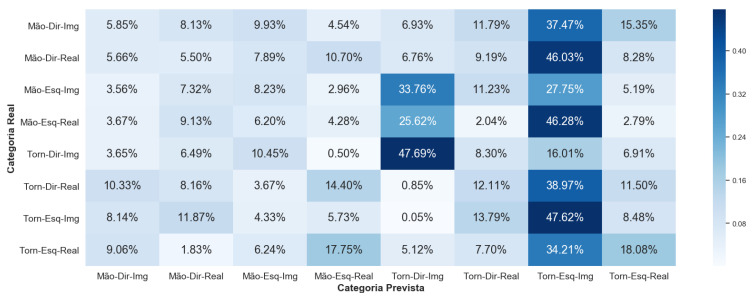
Confusion matrix of Framework 3 using the sensor V-AMP.

**Figure 25:**
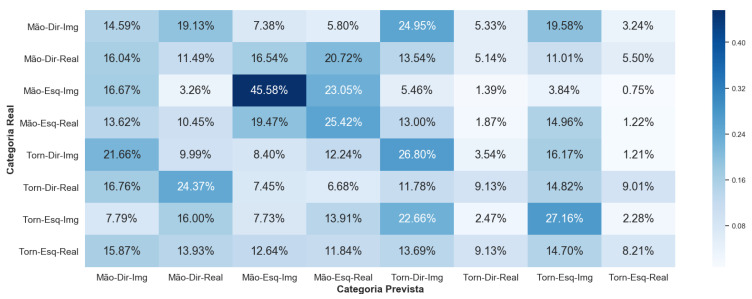
Confusion matrix of Framework 3 using the sensor Mindwave.

Framework 3 seeks to increase the level of difficulty of classification. In addition, it aims to attest to the problems already highlighted by other research groups in the area, which point out that the extraction of characteristics from the EEG signals is a complex process due to the interpersonal variability of the signals. The result for V-AMP data is shown in Figure 24, and for Mindwave in Figure 25. For these graphs, we calculated the average confusion matrix among all individuals.

For this Framework, the performance of the sensors V-AMP and Mindwave were very different and did not follow any logical line. The best but unsatisfactory classification with the V-AMP data was for the imagery left ankle movement. With the data from Mindwave, the image movement of the left-hand was the best.

### 5.3. Comparison between Sensors

The results presented in each framework also indicate that even with different hardware configurations, the devices used in this research could reproduce, in general, consistent results.V-amp as a research device presents superior data quality, and Mindwave even with channel limitations can support specific research applications.

## 6. Conclusions and Final Remarks

The methodology developed in this paper considered implementing a machine learning algorithm with the Random Forest technique to classify real and imagery activities. The set of solutions used converged to satisfactory results.

Both devices, Mindwave and V-AMP, can be used to acquire electroencephalographic signals. However, Mindwave’s data restricts the classification capacity of machine learning for Frameworks 2 and 3. Nevertheless, Mindwave has lower performance than V-AMP since this device was initially conceived for game application and has only one dry electrode positioned in the forehead. Furthermore, more experiments are needed to test the Mindwave applicability in BCI field. Also, the signal quality of the Mindwave sensor might need to be further improved to reduce artifacts.

The implemented algorithm is efficient in distinguishing and classifying motor and imagery activities, especially when the data come from the same subject for the same collection, i.e., Framework 1. The results of Frameworks 2 and 3 indicate that dealing with this type of signal is complex when it comes to collections of the same individual at different times and collections of different individuals at different times. Considering this last point, one of the most significant difficulties is the interpersonal variability of EEG data. However, the proposed algorithm is helpful in BCIs applications where it is possible to perform training and validation processes in each use.

The answer to the questions in the Section 2 are:

- Does ML based on random forest algorithm classify different motor tasks (real or imagery)?

This paper showed that the EEG signals have different characteristics, considering the same subject for a given set of activities. The signal is variable over time, and its characterization process is complex. Nevertheless, ML based on random forest, such as the algorithm implemented in this work, is efficient in classifying these signals, as the results presented in Framework 1.

- Does ML based on random forest differentiate real and motor imagery in FP1 electrode?

It achieved satisfactory performance for Framework 1, with accuracies above 94%. However, for the other frameworks, the classification was not efficient. Therefore, these results indicate that FP1 position can be explored in research that uses the exact data for the entire training and classification process, considering the implementation of machine learning algorithms.

- What is the classification performance comparing consumer-graded and research EEG devices?

Mindwave has a lower performance when compared to V-AMP. However, it was expected that Mindwave would present lower classification accuracy. Nevertheless, results for Framework 1 supports that a consumer-graded EEG could be used in applications involving EEG data classification.

- What is the variability between EEG’s spectro-temporal and spatial distribution characteristics among subjects?

The results showed that EEG data presents temporal variability among the subjects. The best result achieved with the proposed methodology and algorithm was for Framework 1, which considered data from the same individual for the same task. However, this fact does not necessarily indicate that this is the only possibility of classification. There are projections of improvements in the algorithm that can provide the classification process with the ability to identify statistics in the signal of tasks performed by different people—suggestions for further research.

## Figures and Tables

**Table 1:** Summary of key research papers on EEG signal analysis.

Reference	Motor Imagery	Data Acquisition	ML Technique
Amin et al. [16]	Yes	Public dataset	Convolutional Neural Networks
Banu et al. [22]	No	Mindwave	-
Girase et al. [23]	Yes	Mindwave	-
Lazurenko et al. [19]	Yes	EEG monopolarly from 13 standard leads	Neural Networks
Liu et al. [18]	Yes	BioSemi	Random Forest
Meziani et al. [20]	Yes	32 active dry electrode kit	Random Forest, K-Nearest Neighbors, Neural Networks, Support Vector Machines and Linear Discriminant Analysis
Rieiro et al. [21]	No	Mindwave and SOMNOwatch	-
Sadiq et al. [17]	Yes	Public dataset	Support Vector Machines
Siswoyo et al. [24]	No	Neurosky mindset	Neural Networks

**Table 2:** Feature Selection Results for V-AMP data.

Machine	Moments	Channels
A	2 and 4	Fp2 and F4
B0	6	C3 and O2
B1	2, 4 and 6	F3 and P3
C0	3, 5, 7, 8, 9 and 10	Cz, C4 and Pz
C1	4, 6, 8 and 10	F3 and F4
C2	8	O2
C3	2, 4, 6, 8 and 10	P3, Pz, P4, O1 and O2

**Table 3:** Feature Selection Results for Mindwave data.

Machine	Moments
A	2, 3, 4, 5, 6 and 7
B0	2 e 4
B1	2, 3, 4, and 6
C0	2, 3, 4, 5, 6, 7 and 8
C1	2, 4, 5, 7, and 9
C2	6 and 8
C3	2, 4, 6, 7 and 8

## Data Availability

The data of this study are available from the corresponding author upon reasonable request.
